# Maxillary Sinus Ameloblastoma in Turner Syndrome: A Comprehensive Case Report and Long-Term Follow-Up

**DOI:** 10.7759/cureus.47808

**Published:** 2023-10-27

**Authors:** Abdelhak Maghous, Issam Lalya, Hassan Sifat

**Affiliations:** 1 Department of Radiotherapy, Hassan II University of Casablanca, Rabat, MAR; 2 Department of Radiotherapy, Military Hospital Mohamed V, Rabat, MAR

**Keywords:** maxillectomy, radiotherapy (rt), turner syndrome, ameloblastoma, maxillary sinus

## Abstract

This article was previously posted to the Research Square preprint server on 16 August 2023.

Maxillary sinus ameloblastoma is an uncommon, locally aggressive odontogenic tumor. In this case report, we present a comprehensive long-term follow-up of maxillary sinus ameloblastoma occurring in a patient with Turner syndrome, managed through partial right maxillectomy followed by adjuvant operative bed radiotherapy.

## Introduction

This article was previously posted to the Research Square preprint server on 16 August 2023 [14].

Ameloblastoma is an uncommon epithelial odontogenic tumor that originates in the jaw. It is typically large, disfiguring, and locally aggressive, although metastasis is rare. While ameloblastoma predominantly arises in the mandible, its occurrence in the maxillary sinus is less frequent [1-3]. Management of ameloblastoma often entails open maxillectomy, with recurrence being common if resection is inadequate [4-8].

Turner syndrome results from the complete or partial absence of an X chromosome, manifesting as a genetic condition. While the possibility of an elevated cancer risk in this population has been suggested, comprehensive studies are lacking [9-13]. This report presents an unusual case of maxillary sinus ameloblastoma occurring in a patient with Turner syndrome.

## Case presentation

**Figure 1:**
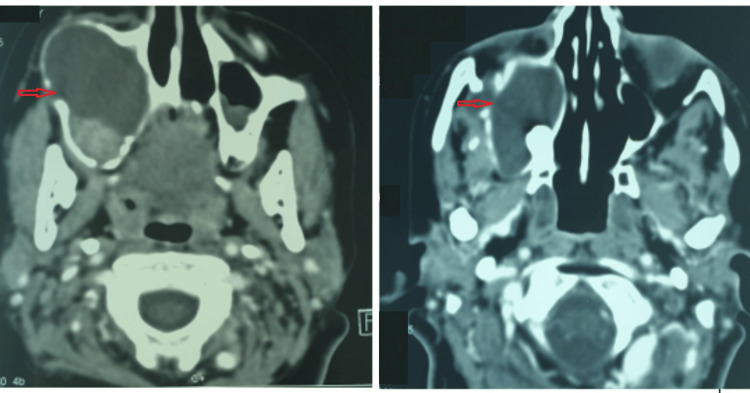
Axial CT showing a massive cystic lesion invading the entire right maxillary sinus with osseous erosion Red arrow: Cystic lesion invading the entire right maxillary sinus

**Figure 2:**
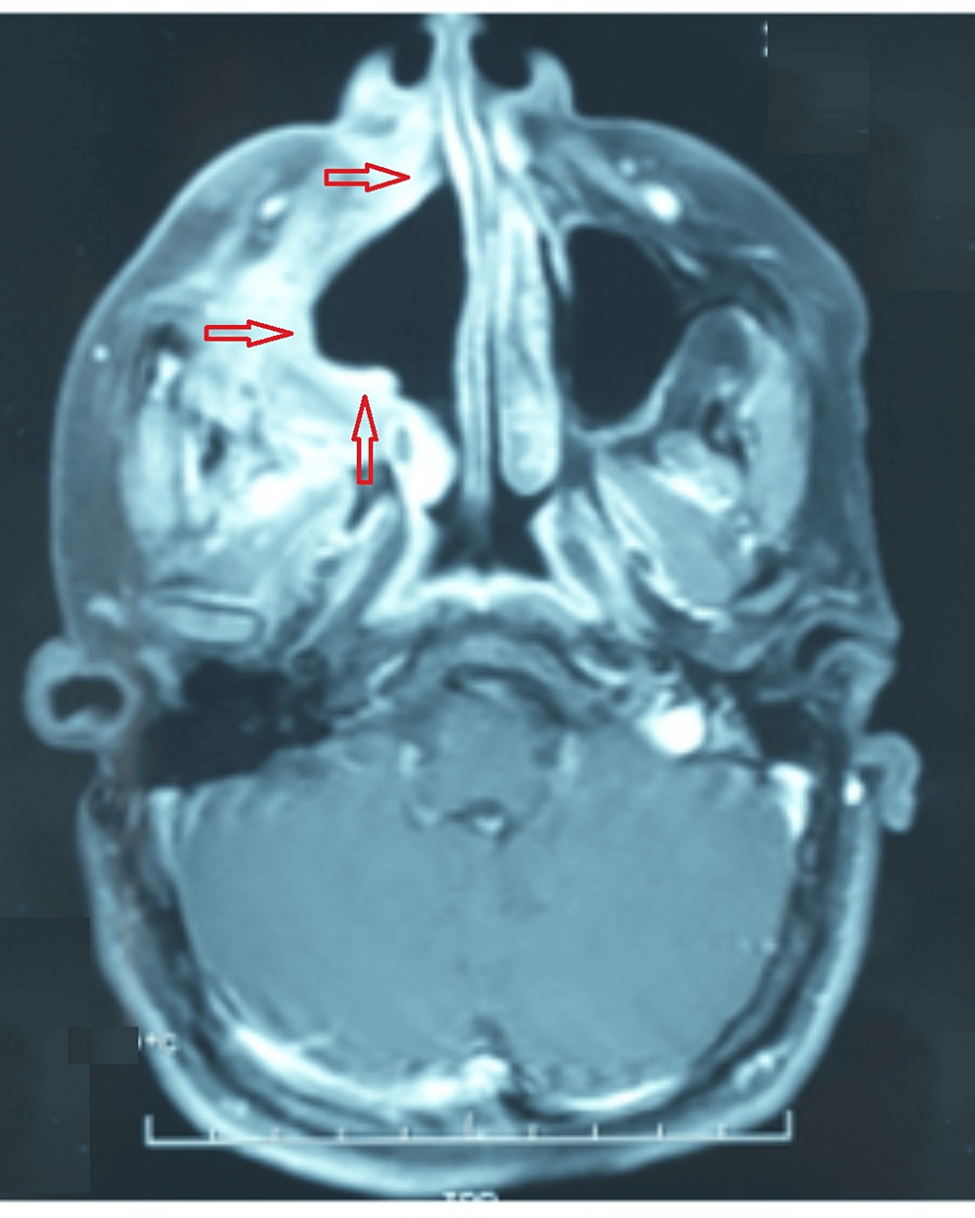
Axial postoperative MRI showing surgical stigmata with residual tissue thickening MRI: Magnetic resonance imaging, Red arrow: Surgical stigmata

**Figure 3:**
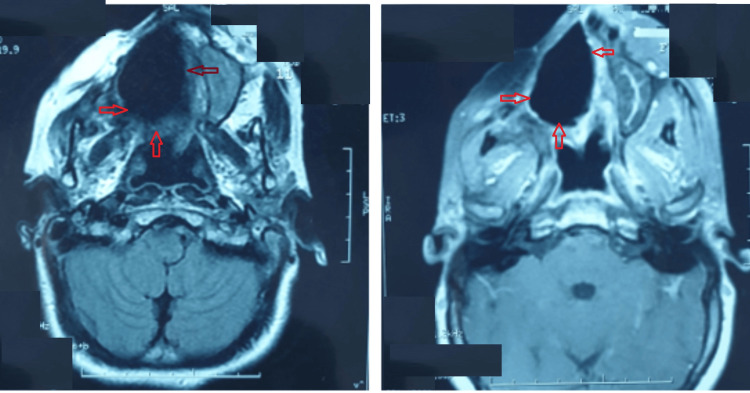
Axial MRI showing stigmata of partial right maxillectomy without progressive lesion after more than 11 years of follow up MRI: Magnetic resonance imaging, Red Arrow: Stigmata of partial right maxillectomy

A 26-year-old woman with Turner syndrome presented to our hospital due to recurrent sinusitis symptoms and an enlarging mass on the right cheek. Computed tomography (CT) of the paranasal sinuses revealed a substantial cystic lesion invading the entire right maxillary sinus, causing osseous erosion (Figure 1). An incisional biopsy was conducted under local anesthesia, and histopathologic examination confirmed plexiform ameloblastoma. Treatment involved a partial right maxillectomy, with the resected specimen validating the ameloblastoma diagnosis but indicating close surgical margins. Subsequent postoperative MRI demonstrated surgical changes, including residual tissue thickening and infiltration into the cutaneous, subcutaneous spaces, and residual muscle planes of the cheek area (Figure 2). Multidisciplinary consultation led to the recommendation of adjuvant radiotherapy. Volumetric modulated arc therapy (VMAT) was utilized to deliver seventy (70) Gy in 35 fractions to the operative bed. Follow-up spanning eleven (11) years displayed no clinical or radiological signs of recurrence (Figure 3). The patient, currently 37 years old, leads a normal life.

## Discussion

Ameloblastoma, an uncommon epithelial odontogenic tumor, constitutes approximately 1% of benign jaw tumors and cysts [3]. This tumor behaves aggressively, gradually invading adjacent tissues in an asymptomatic, painless manner [4-6]. Maxillary sinus ameloblastomas are less common than their mandibular counterparts and are presumed to exhibit greater aggressiveness, with a 50% recurrence rate within five years of initial resection [7]. Optimal treatment entails wide resection into healthy tissues, ensuring safe margins and immediate reconstruction to prevent local relapse [8]. In this case, the patient underwent a partial right maxillectomy, followed by adjuvant radiotherapy targeting the operative bed, effectively mitigating the risk of recurrence posed by the close surgical margins. Our extended follow-up, exceeding 11 years, aligns with literature findings regarding the tumor's low relapse rates [3]. 

Turner syndrome (TS) constitutes the most prevalent sex chromosome abnormality in women, occurring in about 1 in 2500 live female births and typically presenting with retarded growth, short stature, and gonadal dysgenesis [9]. While the possibility of an elevated cancer risk in TS patients has been raised, comprehensive studies are scarce. Schoemaker et al. [10] and Hasle et al. [11], in a multicenter study encompassing numerous TS patients, reported 3.5% and 2.1% neoplasia prevalence in Denmark and Great Britain, respectively. Another TS cohort exhibited a higher tumor prevalence (19.5%) compared to the aforementioned data, with the study indicating a notable incidence of central nervous system (CNS) and gonadal tumors, as well as skin tumors [12]. Pier et al. also suggested an increased neoplasia risk, particularly for multiple meningiomas and skin tumors [13].

## Conclusions

In conclusion, this case report presents a remarkable association between a rare maxillary sinus ameloblastoma and Turner syndrome, challenging our understanding of these distinct medical conditions. This remarkable case emphasizes the importance of early detection, interdisciplinary collaboration, and long-term monitoring for rare tumors, particularly in specific patient populations. While shedding light on potential genetic or environmental factors underlying this association, it underscores the need for further research and a deeper understanding of the complex interplay between these conditions to improve patient care and outcomes.
